# 

**Published:** 1816-11

**Authors:** 


### CORRESPONDENCE.

Mr. Mortimer's excellent Report on the Bulam Fever is received, and will be commenced in our next; Mr. Bailey's Case of enlarged Liver will also appear in our next Number.

Dr. Polydore's remarkable Case of Oneirodynia, with Observations, is received, and will shortly appear. Dr. Johnson's Case of Syncope Angens is postponed for want of room; it will appear in the February Number of our Journal.—Case of Debility removed by Bleeding—of Inflammation of the Stomach, and several other anonymous favours, will be reserved for a particular head, entitled the "PORTE FEUILLE," in the January and succeeding Numbers.—Dr. Breume's Cases of Puerperal Fever have been received, and shall have an early insertion.

☞ Authors and Publishers are requested to send Copies of new Works for Review as early as possible after their publication.

